# Research Progress on MRI for White Matter Hyperintensity of Presumed Vascular Origin and Cognitive Impairment

**DOI:** 10.3389/fneur.2022.865920

**Published:** 2022-07-07

**Authors:** Fanhua Meng, Ying Yang, Guangwei Jin

**Affiliations:** ^1^North China University of Science and Technology, Tangshan, China; ^2^Department of Radiology, China Emergency General Hospital, Beijing, China

**Keywords:** white matter hyperintensities of presumed vascular origin, cerebral small vascular disease, cognitive impairment, magnetic resonance imaging, neuroimaging, white matter

## Abstract

White matter hyperintensity of presumed vascular origin (WMH) is a common medical imaging manifestation in the brains of middle-aged and elderly individuals. WMH can lead to cognitive decline and an increased risk of cognitive impairment and dementia. However, the pathogenesis of cognitive impairment in patients with WMH remains unclear. WMH increases the risk of cognitive impairment, the nature and severity of which depend on lesion volume and location and the patient's cognitive reserve. Abnormal changes in microstructure, cerebral blood flow, metabolites, and resting brain function are observed in patients with WMH with cognitive impairment. Magnetic resonance imaging (MRI) is an indispensable tool for detecting WMH, and novel MRI techniques have emerged as the key approaches for exploring WMH and cognitive impairment. This article provides an overview of the association between WMH and cognitive impairment and the application of dynamic contrast-enhanced MRI, structural MRI, diffusion tensor imaging, 3D-arterial spin labeling, intravoxel incoherent motion, magnetic resonance spectroscopy, and resting-state functional MRI for examining WMH and cognitive impairment.

## Introduction

With the growth in aging populations worldwide and the high incidence of risk factors for cerebrovascular diseases, comorbid neurodegenerative diseases such as Alzheimer's disease (AD) and cerebrovascular diseases are common, resulting in a heavy burden on families and society (1). White matter hyperintensity of presumed vascular origin (WMH) is an imaging marker of cerebral small vessel disease (CSVD) and its common imaging manifestation in the brains of middle-aged and elderly individuals (2, 3). The reported prevalence of WMH varies due to the differences in patient's characteristics, imaging techniques, and rating methods (4). The prevalence of WMH ranges from 39 to 100% and increases with age (5). Of individuals aged 60–70 years, 87% have deep WMH (DWMH), and 68% have periventricular WMH (PVWMH), whereas of individuals aged 80–90 years, 100% have DWMH, and 95% have PVWMH (5). Several factors contribute to the formation of WMH, including age, hypertension, apolipoprotein E ε4 allele, diabetes, hyperlipidemia, race, female sex, smoking, and alcohol consumption (5, 6). Studies have demonstrated that WMH is associated with cognitive decline, dementia, depression, stroke, gait disorder, and urinary system problems (5, 6).

Extensive evidence indicates that WMH is associated with cognitive impairment (4, 7, 8). WMH increases the risk of all-cause dementia, AD, and vascular dementia by 14, 25, and 73%, respectively (8). Notably, the nature and severity of related cognitive impairment depend on the volume and location of WMH and the patient's cognitive reserve (4, 7). Compared to conventional MRI, the application of novel MRI techniques to explore the relationship between WMH and cognitive impairment may facilitate early diagnosis of WMH and predict its progression, which is critical to enhancing our understanding of the pathogenesis of WMH and associated cognitive impairment.

## WMH

### Definition of WMH

Before 2013, the medical imaging diagnostic terms for WMH were not unified. Wardlaw et al. (2) summarized the terms used in WMH, including leukoaraiosis, white matter lesions, white matter hyperintensity, leukoencephalopathy, and white matter disease. Standards for Reporting Vascular Changes on Neuroimaging (STRIVE) recommended the term “white matter hyperintensity of presumed vascular origin” and defined WMH in 2013. WMH is hyperintense on T_2_-weighted and T_2_-fluid attenuated inversion recovery sequences; it may appear as isointense or hypointense (less hypointense than cerebrospinal fluid) on T_1_-weighted image (T_1_WI) sequences, depending on the severity of pathological changes and sequence parameters. Notably, lesions of the subcortical gray matter and brainstem should not be classified as WMH, and subcortical hyperintensities can be used as an alternative collective term (2).

### Possible Pathogenesis of WMH

The pathogenesis of WMH is complex and multifactorial. Currently, WMH pathogenesis remains unclear. The pathological manifestations of WMH include demyelination, oligodendrocyte apoptosis, axonal damage, and gliosis (9, 10). Several acquired risk factors (such as age and hypertension) interact with WMH susceptibility caused by congenital risk factors (such as the apolipoprotein Eε4 allele), resulting in arterial and venous diseases. Cerebral arteriolar stenosis, arteriosclerosis, and endothelial dysfunction lead to diffuse loss of dynamic cerebral autoregulation.

In contrast, venous collagenosis, such as venous ischemia, periventricular small vein collagen deposition, jugular vein reflux, and pulse wave encephalopathy, causes venous reflux limitation and leads to venous hypertension. The loss of dynamic cerebral autoregulation and venous collagenosis can lead to white matter hypoperfusion, blood–brain barrier (BBB) damage, and ependyma damage. Further, hypoperfusion causes white matter ischemia and damage to the BBB and ependyma, resulting in the leakage of plasma or cerebrospinal fluid components into the brain parenchyma. These cause inflammation and apoptosis, demyelination, oligodendrocyte apoptosis, axonal injury, and gliosis, which may eventually lead to WMH (9, 10).

## WMH and Cognitive Impairment

### WMH Volume and Cognitive Impairment

White matter hyperintensity of presumed vascular origin volume is associated with cognitive impairment, whereby a larger WMH volume is associated with a greater decline in cognitive function (4, 8). Patients with confluent WMH have a greater decrease in annual Mini-Mental State Examination scores; patients with mild cognitive impairment (MCI) and confluent WMH are more likely to develop AD (11). Moreover, WMH volumes are significantly increased in patients with MCI that evolve to AD (12). Studies on the relationship between total WMH volume and cognitive impairment have yet to reach a consensus. Some scholars believe that total WMH volume is negatively correlated with executive function, memory, and speed performance (5, 13, 14). However, Melazzini et al. (15) propose that there is no correlation between total WMH volume and cognitive ability. The presence of WMH does not necessarily lead to cognitive impairment, indicating that patients with WMH may be asymptomatic (16). Decarl highlighted that WMH volume was associated with decreased cognitive scores only when >0.5% of the total intracranial volume (17). These findings suggest that WMH volume is closely associated with cognitive impairment. Nevertheless, the relationship between total WMH volume and cognitive impairment requires further investigation.

### WMH and Cognitive Impairment in Different Locations and T_1_WI Signal Intensity

White matter hyperintensity of presumed vascular origin location is associated with specific cognitive impairment and spatial specificity (7, 8). The correlation between PVWMH and cognitive impairment is stronger than that between PVWMH and DWMH. PVWMH is associated with various types of cognitive impairment, whereas DWMH is negatively correlated with motion speed (8, 14, 15). A possible pathogenic mechanism involves PVWMH-mediated disruption of long-distance white matter connectivity, leading to cognitive decline in several locations. DWMH disrupts short-distance connections and impairs cognitive abilities in specific brain regions (18, 19). One study reported that frontal WMH near the frontal ventricles affected executive function and parieto-temporal WMH near the posterior horns resulted in memory deterioration (14). Kaskikallio et al. demonstrated that the parieto-occipital region was associated with processing speed and speech memory disorders, whereas WMH in the upper deep white matter compromised motor speed performance. Temporal lobe WMH is associated with processing speed impairment, and the processing speed of patients with MCI and AD with temporal lobe WMH is significantly decreased (20).

Recently, Luca et al. (15) employed a novel classification method to classify WMH into four types: T_1_-hypointense PVWMH, non-T_1_-hypointense PVWMH, T_1_-hypointense DWMH, and non-T_1_-hypointense DWMH. They reported that WMH with T_1_WI hypointensity was associated with poorer cognitive ability, and WMH with T_1_WI hypointensity around the ventricle was significantly associated with cognitive impairment. These results suggest that PVWMH is more closely associated with cognitive impairment than DWMH and that WMH in different brain regions is associated with distinct cognitive impairment. WMH with T_1_WI hypointensity may be the most severe WMH. In this regard, combining the traditional classification of WMH with the location and intensity of lesions in T_1_WI may be more valuable than previous classification approaches (12, 15).

### Changes in WMH and Cognitive Impairment

White matter hyperintensity of presumed vascular origin may progress or regress; these processes may even occur simultaneously (4, 10, 21). The volume and severity of WMH increase over time (4, 5). One study showed that 25.5% of participants had regression of WMH, 19.1% had no change in WMH, and 55.4% had progression of WMH (22). In the 2008 Rotterdam Scan Study, 39% of participants had increased WMH volume within 3.4 years. In the 2009 Oregon Brain Aging Study, 84% of patients developed WMH progression within 9.1 years. Overall, the annual growth rate of WMH volume was 4.4–37.2% (5). When patients with MCI evolved to AD, the T_1_WI hypointense intensity of paracortical WMH was significantly decreased, and the T_1_WI hypointense intensity of other regions decreased significantly with age (12). Large-scale longitudinal MRI studies have demonstrated that increased WMH volume can lead to an accelerated decline in cognitive function (4, 10). The mean annual decline in cognitive function is ~2-folds for each standard deviation increase in WMH volume (13). An increase in PVWMH and DWMH volumes is associated with a decrease in Mini-Mental State Examination scores (4, 23). In addition, an increase in DWMH volume is significantly correlated with a change in language fluency score (23), and an increase in PVWMH volume is correlated with a decrease in information processing speed and general cognitive ability (4).

Studies have also reported a reduction in WMH. In a previous study (24), 71 participants (37%) exhibited a decrease in WMH within 1 year. Another study evaluated middle-aged and elderly participants living in the community three times over 9 years. During the first follow-up, 26 participants (9.4%) exhibited a decrease in WMH volume, but only 1 participant (0.4%) exhibited a decrease in WMH volume throughout the follow-up period (21). A recent study reported that 87 patients with subcortical vascular cognitive impairment had WMH progression, and 17 had WMH regression over a 3-year period (25). The cognitive function of the two groups decreased, and there was no difference in the rate of decline of language, visuospatial function, memory, executive function, or general cognitive function. These results may be due to the severe WMH burden at baseline and the small sample size of the study. Although the net amount of WMH was generally reduced in the WMH regression group, severe WMH may have perturbed network connections and continued to progress, resulting in a significant decline in cognitive function (25). These findings suggest that the changes in WMH are non-linear, which can accelerate over time, or their progress may be attenuated or interrupted for various reasons (21). In summary, WMH progression can lead to an accelerated decline in cognitive ability.

## Application of Multimodal MRI Techniques for WMH and Cognitive Impairment

### Structural Magnetic Resonance Imaging

White matter hyperintensity of presumed vascular origin, cognitive impairment, and brain atrophy often coexist in older adults. Voxel-based morphometry enables quantitative measurement of the volume of whole-brain white matter and gray matter and accurate analysis of the difference in volume and density of gray matter. Gray matter defects are associated with reduced cognitive function in patients with WMH (26). A recent study demonstrated that WMH was associated with a decrease in gray matter in the middle temporal gyrus, right medial frontal gyrus, and left parahippocampal gyrus (27). This agrees with another study that reported significantly lower cortical and subcortical widespread gray matter density in participants with WMH with cognitive impairment/AD than in control participants; Moreover, white matter volume was significantly different between participants with WMH with cognitive impairment/AD and the control group (26). In addition, Ashwati's results revealed non-unidirectional relationships between WMH burden, gray matter volume, and cognition in MCI. At a high burden, WMH and gray matter volume were negatively correlated, whereas at a low burden, WMH and gray matter volume were positively correlated. The negative correlation between WMH and memory and executive function is regulated by regional gray matter volume (28).

Previous studies have demonstrated that cortical thinning and WMH are associated with cognitive impairment (29, 30). Longitudinal studies have revealed that parietal WMH is associated with left entorhinal cortical and right frontal atrophy, and total WMH volume is associated with cortical thinning in the right frontal and parietal regions. Moreover, cortical thinning is associated with poorer memory (29), and WMH progression is associated with faster cortical thinning (25). These results suggest that WMH may promote brain atrophy, leading to cognitive decline. These data may deepen our understanding of the pathogenesis of cognitive impairment in patients with WMH.

### Dynamic Contrast-Enhanced Magnetic Resonance Imaging

Blood–brain barrier dysfunction is one of the pathophysiological mechanisms of WMH (9, 10). Dynamic contrast-enhanced magnetic resonance imaging (DCE-MRI) is widely used as an indicator of WMH, as well as for assessing the functional integrity of the BBB (31). Studies have shown that BBB leakage was greater in patients with WMH than in those without WMH (32, 33). Among participants with higher WMH load, the leakage rate of BBB in normal-appearing white matter (NAWM), WMH, and gray matter was significantly higher (31), and there was no significant difference in the leakage rate of NAWM, WMH, and cortical gray matter (33). Another study found that a lower leakage rate in WMH was associated with a larger WMH volume. This may be due to the decreased perfusion of WMH, which needs further study (34). BBB leakage increased with increased hypertension and the load of WMH and NAWM (35). Higher BBB permeability is associated with higher WMH burden and decreased cognitive ability (31). Higher BBB leakage at baseline was associated with stronger cognitive decline, especially in executive function (32). BBB dysfunction may be the mechanism of CSVD and cognitive impairment. The increase of BBB leakage in the WMH area indicates the deterioration of cognitive function in the future (31, 33, 36). These results suggest that DCE-MRI may help to evaluate the permeability of BBB in WMH and its relationship with cognitive impairment. Impaired BBB integrity may be a key factor in the pathogenesis of WMH and part of a series of pathological processes that eventually lead to cognitive impairment.

### Diffusion Tensor Imaging

Diffusion tensor imaging is widely used to evaluate the microstructural integrity of the matter. Common DTI parameters include fractional anisotropy (FA), mean diffusivity (MD), axial diffusivity, and radial diffusivity (37). It is generally believed that lower FA and higher MD reflect poor microstructural integrity of white matter (38, 39). Cognitive function is closely associated with white matter integrity detected by DTI (40, 41). The related injury is not limited to visible lesions but also exists in NAWM around the WMH. Over time, abnormal changes in NAWM precede the progression of WMH, referred to as the WMH penumbra (42). In a study by Zhong et al. (43), patients with WMH underwent DTI and 3D-arterial spin labeling (3D-ASL). The results demonstrated that FA and MD of NAWM were significantly correlated with WMH volume, and multiple linear regression analysis indicated that overall cognitive function was independently correlated with WMH-FA and NAWM-FA but not with cortical cerebral blood flow (CBF). This suggests that the relationship between overall cognitive function and white matter integrity may be closer than that with blood supply.

Cognitive impairment in patients with WMH is associated with the microstructural destruction of various white matter fibers, which may include “disconnection” of cortical-subcortical pathways (40, 41). Yuan et al. (40) reported that compared with that in NAWM and control groups, FA in the WMH group was significantly decreased, and MD was significantly increased. The MD values of the periventricular white matter and corpus callosum in the NAWM group were significantly higher than those in the control group, suggesting the destruction of the nerve fiber bundles. A recent study (39) demonstrated that in patients with WMH, the MD values of several fiber bundles, including the bilateral anterior thalamic radiation, left inferior fronto-occipital fasciculus, right inferior longitudinal fasciculus, and right superior longitudinal fasciculus, were negatively correlated with memory function. The anterior part of the right inferior fronto-occipital fasciculus and the posterior and middle parts of the right inferior longitudinal fasciculus were negatively correlated with Mini-Mental State Examination scores and episodic memory. These results suggest that WMH pathogenesis may be related to the microstructural integrity of the white matter. In this regard, cognitive impairment in patients with WMH may be due to the “disconnection” of cortical-subcortical pathways.

### 3D-ASL

Cerebral perfusion decreases throughout the life cycle and changes in the early stages of age-related neuropathies (44). 3D-ASL permits non-invasive quantification of CBF and has good reliability and repeatability (45). One study reported that greater WMH severity was associated with lower average CBF of whole-brain white matter and CBF at and around the lesion (42, 46). A quantitative study of CBF in patients with different degrees of WMH using 3D-ASL revealed that CBF in WMH was lower than that in the surrounding tissues, and whole-brain CBF in patients with confluent WMH was 20% lower than that in patients with spotted or newly confluent WMH (47). CBF is significantly reduced in patients with cognitive impairment (48). A longitudinal study reported that a decrease in whole-brain CBF was associated with a decrease in processing speed, and better baseline perfusion was associated with better executive function. A decrease in whole-brain perfusion is also associated with deterioration of brain structure and a decrease in processing speed (44). Brain perfusion may significantly impact the maintenance of white matter integrity in patients with WMH (43). Patients with MCI with WMH exhibit reduced regional cerebral blood flow in the frontal, parietal, and medial temporal lobes and putamen compared to those without WMH (49). The decrease in CBF is related to the volume of WMH (50). Among patients with AD, patients with WMH have less local CBF and a wider range than patients without WMH, especially in the frontal and middle temporal lobes (51). Crucially, a larger volume of WMH is associated with lower whole-brain and cortical CBF (50, 52). In a study on patients with AD and control patients without AD (53), whole-brain CBF and CBF in different brain regions were significantly reduced in the AD group. Meanwhile, whole-brain CBF, PVWMH, and DWMH were positively correlated with Montreal Cognitive Assessment Scale scores.

Arterial spin labeling permits the detection of CBF changes in the penumbra (42). A study compared the penumbra of WMH structure with that of CBF using FLAIR, pulsed arterial spin labeling, and DTI. The variation in DTI parameters extended to 2–9 mm around the WMH, whereas the variation in CBF extended to 13–14 mm. This suggests that the CBF penumbra may be more extensive than the structural penumbra in WMH tissues, with or without microstructural changes (54). Collectively, these results suggest that WMH severity is negatively correlated with CBF, which may contribute to the early diagnosis and prediction of WMH. These data provide help to the pathogenesis of WMH and cognitive impairment in patients with WMH.

### Intravoxel Incoherent Motion

Intravoxel incoherent motion enables the simultaneous evaluation of microvasculature and microstructure. Accordingly, this approach provides insight into the interplay between brain tissue and vessels. Moreover, it does not rely on tracer delivery (55, 56). IVIM has revealed increased parenchymal diffusivity and decreased perfusion in patients with CSVD and a correlation between cognitive decline and WMH (56). Sun et al. (55) demonstrated that both PVWMH and DWMH are associated with decreased fast diffusion and increased slow diffusion. An increased perfusion fraction in PWMH is associated with improved cognitive function. The observed association between decreased microvascular perfusion of NAWM and decreased cognitive function supports previous findings that NAWM in CSVD may be affected before pathological abnormalities (i.e., WMH) become apparent and perfusion abnormalities may precede structural abnormalities (57).

Nevertheless, this result is not fully consistent with previously reported results. One study demonstrated that both perfusion volume fraction and parenchymal diffusivity were higher than those in the control group and increased with an increase in WMH burden (58). Further research is warranted to clarify the relationship between perfusion volume fraction and blood flow. Nevertheless, findings from IVIM imaging indicate that increases in perfusion fraction and parenchymal diffusivity in WMH are both associated with disease severity, highlighting the potential of IVIM imaging as a surrogate marker for CSVD.

### Magnetic Resonance Spectroscopy

Magnetic resonance spectroscopy technology is an MR technology used for the non-invasive evaluation of metabolic changes in brain tissue. 1H-MRS is currently the most widely used method. Its main metabolites are N-acetylaspartate, choline, creatine, and phosphocreatine (59). Reports suggest that cognitive function in patients with WMH is associated with neurometabolite levels (60). One study reported that in patients with vascular cognitive impairment, creatine was significantly correlated with executive function, memory, attention, and overall cognitive scores; N-acetylaspartate was significantly correlated with executive function and overall cognitive scores; changes were observed in metabolic concentrations in both WMH and NAWM (61). The assessment of neurometabolite levels in patients with WMH provides additional information about vascular cognitive impairment and cognitive function, which may not be readily available by measuring WMH volume (61). Another study (62) reported that the N-acetylaspartate/creatine and N-acetylaspartate/choline ratios in WMH were significantly decreased, and cognitive function score was positively correlated with N-acetylaspartate/choline and N-acetylaspartate/creatine ratios in WMH and N-acetylaspartate choline ratios in NAWM. In addition, Xing et al. (60) proposed that the N-acetylaspartate/creatine and choline/creatine ratios in 1H-MRS can be used to diagnose early WMH and evaluate cognitive impairment in patients with WMH. In sum, MRS is a useful technique for the early diagnosis of WMH and cognitive impairment, which may help to elucidate WMH pathogenesis.

### Resting-State Functional Magnetic Resonance Imaging

Resting-state functional magnetic resonance imaging reflects the activity of brain regions by relying on MR signals generated by the changes in the blood oxygen levels of brain tissues in the resting state (63). The oxygen extraction fraction (OEF) is an important parameter of brain metabolism and a key biomarker of tissue vitality, detecting oxygen utilization rate to oxygen delivery rate (64). Compared with patients without WMH, patients with WMH have significantly lower CBF values and significantly higher OEF values (65, 66). This may be due to increased oxygen extraction due to abnormally reduced blood flow (67). With the increase of WMH density, CBF decreased, and OEF increased. In addition, in concentric contours close to WMH, OEF gradually increased, and CBF decreased (66). WMH is related to OEF, but it will change due to the existence of cognitive impairment (68). The lower the OEF, the more severe the cognitive impairment, possibly due to reduced oxygen consumption resulting from reduced neural activity (67). The recent studies have shown that WMH is related to OEF, but it will change due to the existence of cognitive impairment (68). The OEF of cognitively impaired subjects was higher than that of those with cognitive integrity. The increase of WMH load in cognitive impairment subjects was significantly correlated with the decrease of OEF, but not in the cognitively intact (68).

The amplitude of low-frequency fluctuations (ALFF) of rs-fMRI signals can be used to detect spontaneous brain activity under physiological conditions. ALFF has been used to study WMH and associated cognitive impairment (69, 70). Cognitive impairment in patients with WMH may be associated with different amplitude fluctuations in rs-fMRI signals (69). A previous study (71) reported the large differences in ALFF predominantly in the posterior cingulate cortex, posterior precuneus, and right inferior temporal gyrus. The ALFF value of the inferior temporal gyrus was significantly higher in the WMH-MCI group than in the WMH-AD and control groups, and the change in ALFF was positively correlated with the executive function score. Moreover, the ALFF value of the temporal posterior cingulate cortex was significantly lower, and the ALFF value of the precuneus was significantly higher in patients with WMH-AD than in the control group. Another study (69) demonstrated that ALFF values in the right inferior occipital gyrus, left precuneus, right superior frontal gyrus, and right superior occipital gyrus were significantly higher in the non-cognitive WMH group than in the normal control group. Further, the ALFF values of the right inferior occipital gyrus, superior occipital gyrus, left middle temporal gyrus, and precuneus were significantly lower in the cognitively impaired WMH group than in the non-cognitively impaired WMH group.

Cognitive impairment in patients with WMH may be associated with abnormal functional connectivity (FC) (72, 73). In one study (73), FC in subcortical nuclei and central cortical areas of cognitive networks was decreased in patients with WMH-MCI, especially in the cingulate cortex. Another study reported that (72), compared with the control group, the MCI group exhibited decreased FC between the precuneus seeds and bilateral lateral temporal cortex, medial prefrontal cortex, posterior cingulate cortex, and parietal lobe. There was a significant regional correlation between WMH volume and default mode network (DMN) FC in the MCI group. FC of the DMN is lower in patients with MCI than in cognitively healthy elderly, and its degree is distinct from that of WMH. These findings suggest that WMH plays a key role in the destruction of DMN in patients with cognitive impairment. In a recent study by Chen et al. (74), participants underwent DTI and rs-fMRI. The results revealed that participants with a higher WMH load had higher FC in the DMN of the medial frontal gyrus and lower FC in the DMN of the thalamus. The increase in radial diffusivity in the white matter bundle between the hippocampus and posterior cingulate cortex was identified as an independent indicator of poor memory. These abnormal FC and structural connections were independently associated with slower processing speed and poor memory. Collectively, these results suggest that OEF, ALFF, and FC obtained by rs-fMRI can be used to explore WMH and cognitive impairment, which may help to deepen our understanding of the pathogenesis of cognitive impairment in patients with WMH.

## Discussion and Conclusion

The pathogenesis of WMH is heterogeneous. Studies have shown that the vascular anatomy and pathogenesis of PVWMH and DWMH are different, but ischemia is both involved (9, 10). It should be noted that the smooth halo hyperintensity adjacent to the ventricle is associated with ependymal rupture, ependymal gliosis, and myelin loss but is not caused by ischemia (75). WMH induced by arterial stenosis usually shows dot or patchy hyperintensity with an obvious dividing line. Arteriosclerosis-related WMH is mainly characterized by symmetrical hyperintensity around the basal ganglia. However, venous white matter lesions may have symmetrical cloud patterns around bilateral periventricular (signal intensity is often lower than WMH). Multiple ischemic foci may be caused by asymptomatic cardiogenic embolism; cerebral amyloid angiopathy-related WMH may be unevenly distributed, but it is more likely to occur in the occipital lobe (76).

In conclusion, WMH is closely associated with cognitive impairment, which is related to the location, size, and progression of WMH. In addition, WMH with T_1_WI hypointensity seems more detrimental than WMH without T_1_WI hypointensity, and the former is closely related to cognitive impairment. Structural MRI research suggests that WMH may accelerate changes in brain structure, promote neurodegenerative changes, and affect cognitive ability. DTI and 3D-ASL are the useful tools for the early clinical diagnosis and prediction of WMH. Pathogenesis identified by DTI may be related to the integrity of white matter microstructure, whereas 3D-ASL enables CBF quantification. CBF abnormalities in patients with WMH precede microstructural changes, which may be due to the integrity of white matter microstructure requires maintenance of CBF. IVIM imaging indicates that the increase in WMH perfusion fraction and parenchymal dispersion is associated with disease severity. In this regard, IVIM imaging may be harnessed as a novel marker of CSVD. MRS can provide additional information through the evaluation of neurometabolite levels in patients, which is useful for the early diagnosis of WMH and cognitive impairment as well as for understanding the pathogenesis of WMH. Rs-fMRI permits the detection of spontaneous brain activity and FC to explore WMH and cognitive dysfunction.

The research is currently limited to single pathogenic mechanisms, and the relationship between different mechanisms should not be overlooked. Simultaneously, treatment measures are limited by research on pathogenesis. In the future, molecular biology can be harnessed as a bridge in combination with multimodal MRI imaging, neurobiology, and other disciplines to further explore the pathogenesis and treatment of WMH and cognitive impairment by expanding clinical sample sizes, increasing follow-up time, and exploring novel biomarkers.

## Author Contributions

FM, YY, and GJ have made a substantial, direct, and intellectual contribution to the work and were involved in the preparation, correction, and modification of the manuscript. All authors contributed to the article and approved the submitted version.

## Funding

This study was funded by Medical Development Scientific Research Fund of China Emergency General Hospital (K202106).

## Conflict of Interest

The authors declare that the research was conducted in the absence of any commercial or financial relationships that could be construed as a potential conflict of interest.

## Publisher's Note

All claims expressed in this article are solely those of the authors and do not necessarily represent those of their affiliated organizations, or those of the publisher, the editors and the reviewers. Any product that may be evaluated in this article, or claim that may be made by its manufacturer, is not guaranteed or endorsed by the publisher.

## Abbreviations

AD: Alzheimer's disease
ALFF: low-frequency fluctuations
BBB: blood–brain barrier
CBF: cerebral blood flow
CSVD: cerebral small vessel disease
DCE-MRI: enhanced magnetic resonance imaging
DTI: diffusion tensor imaging
DWMH: deep WMH
FC: functional connectivity
IVIM: intravoxel incoherent motion
MCI: mild cognitive impairment
MRI: magnetic resonance imaging
MRS: magnetic resonance spectroscopy
NAWM: normal-appearing white matter
OEF: oxygen extraction fraction
PVWMH: periventricular WMH
Rs-fMRI: resting-state functional magnetic resonance imaging
sMRI: structural magnetic resonance imaging
T_1_WI T_1_: weighted image
WMH: white matter hyperintensity of presumed vascular origin
3D-ASL: 3D-arterial spin labeling.
